# The Prognostic Role of Mitral Valve Regurgitation Severity and Left Ventricle Function in Acute Heart Failure

**DOI:** 10.3390/jcm11154267

**Published:** 2022-07-22

**Authors:** Israel Mazin, Michael Arad, Dov Freimark, Ilan Goldenberg, Rafael Kuperstein

**Affiliations:** 1 Heart Institute, Cardiology Division, Sheba Medical Center, Tel-Aviv University, Tel Hashomer, Ramat Gan 5365601, Israel; michael.arad@sheba.health.gov.il (M.A.); dov.freimark@sheba.health.gov.il (D.F.); rafael.kuperstein@sheba.health.gov.il (R.K.); 2 Clinical Cardiovascular Research Center, University of Rochester Medical Center, Rochester, NY 14642, USA; ilan.goldenberg@heart.rochester.edu

**Keywords:** mitral regurgitation, left ventricular ejection fraction, acute heart failure, mortality, heart failure hospitalization

## Abstract

*Aims:* Data about the prognostic interplay between mitral regurgitation MR and left ventricular (LV) function in the outcome of patients admitted with acute heart failure (AHF) are scarce. We evaluated the prognostic impact of MR severity and LV function on mortality and on recurrent heart failure hospitalization (re-HFH) in patients admitted with AHF. *Methods and Results:* In total, 6843 patients admitted with AHF were evaluated: 2521 patients with LV ejection fraction (LVEF) ≤ 40% (reduced LVEF), 1238 of them (51%) having ≥moderate MR; and 4322 with LVEF > 40% (preserved LVEF), 1175 of them (27%) having ≥moderate MR. One-year mortality and re-HFH rates were higher in patients with ≥moderate MR unrelated to the baseline LV function (*p* = 0.028 and *p* < 0.001, respectively). After multivariable analysis, only reduced LVEF, and not the severity of MR, predicted mortality risk (HR: 1.31 [95% CI: 1.12–1.53] for patients with reduced LV function and ≤mild MR; HR: 1.44 [95% CI: 1.25–1.67] for patients with reduced LV function and ≥moderate MR); *p* < 0.001 for both. There was an increased risk for re-HFH in each group (HR: 1.35 [95% CI: 1.17–1.52] for patients with preserved LV function and ≥moderate MR; HR: 1.31 [95% CI: 1.15–1.51] for patients with reduced LV function and mild MR; and HR: 1.65 [95% CI: 1.45–1.88] for patients with reduced LV function and ≥moderate MR); *p* < 0.001 for all. *Conclusions:* In patients admitted with AHF, the LV function is the main prognostic determinant for mortality after 1 year. Significant (≥moderate) MR is associated with an increased risk of recurrent hospitalization.

## 1. Introduction

Mitral regurgitation (MR) is a common finding in patients with heart failure [1] and many studies in chronic heart failure patients have shown that MR is associated with poor outcomes [2,3,4,5]. What remains unclear is which patients’ MR is just a correlate of more advanced cardiomyopathic remodeling, and in which patients, the MR itself directly contributes to the adverse prognosis of these patients through additional LV volume loading [6,7].

In contrast to the extensive literature about the prognostic impact of MR in patients with chronic heart failure [8,9], data about the prognostic interplay between MR severity and LVEF in patients admitted with acute heart failure (AHF) are scarce [10,11,12]. 

The aim of the current study was to evaluate the prognostic impact and the interaction of LVEF and MR severity on one-year mortality and recurrent heart failure hospitalization (re-HFH) in a large group of patients admitted with a diagnosis of AHF to a tertiary center. 

## 2. Methods

This study included all patients admitted with a diagnosis of AHF at the Sheba Medical Center between 2008 and 2016. All patients underwent a full echocardiographic evaluation during the first 2 days of admission at their index hospitalization. All echocardiographic examinations were performed by specialized echocardiography technologists and further analyzed and reported by a specialized echocardiographist.

The left ventricle (LV) and atrial dimensions were measured according to the ASE guidelines for chamber quantification [13]. LVEF was visually estimated. We divided our cohort into 3 groups according to the current ESC guidelines for the diagnosis and treatment of acute and chronic heart failure [14].

The grading of MR severity (mild, moderate, or severe) was performed using a combination of semiquantitative and quantitative assessment, as described by the American Society of Echocardiography guidelines and the European Association of Echocardiography [15,16]. Specifically, mitral regurgitation with narrow central jets, absent PISA radius or an ERO < 0.2 cm^2^, a narrow vena contracta, mitral A wave dominant flow, and a faint, continuous wave Doppler signal were classified as mild mitral regurgitation, while patients with PISA radius > 1 cm^2^, vena contracta width ≥ 0.7 cm, mitral E wave dominant flow, pulmonic venous flow reversal, and a dense triangular, continuous wave Doppler signal or a central jet occupying > 50% of the LA area or eccentric jets adhering to the left atrial wall and reaching the posterior LA wall were classified as severe mitral regurgitation. Patients with intermediate findings were classified as moderate mitral regurgitation. Clinical data regarding rehospitalization rates were obtained through a chart review. Mortality data were obtained from the Israeli Interior Ministry records. The study was approved by the respective human research review board of Sheba Medical Center. Patients were not involved in the planning and designing of this study. 

### Statistical Analysis

Continuous data are presented as mean ± SD and compared using Student’s *t*-test and one-way ANOVA. Categorical data were compared with the use of the chi-squared test or Fisher’s exact test as appropriate. Baseline characteristics, laboratory results, pharmacological treatment, and echocardiography data were compared between patients according to the degree of MR (up to mild or ≥moderate) and then further subdivided according to the LVEF categories during the indexed event. Kaplan–Meier curves were used to calculate the probability of all-cause mortality and recurrent heart failure hospitalization at 1 year for the abovementioned divisions. Multivariable Cox proportional-hazard regression modeling was used to evaluate hazard ratios for the events. Adjustments were made according to clinically important covariates that include demographic status (age, gender, and body surface area (BSA)) and comorbidities (atrial fibrillation, IHD, renal failure (eGFR < 60 mL/min/1.73 m^2^), anemia (hemoglobin below 13 gr/dL at men and 12 gr/dL in women), diabetes mellitus, and COPD). A further subgroup-sensitive analysis (multivariable Cox proportional-hazard regression) for above and below median left ventricular end-diastolic diameter was conducted. We used three different models: unadjusted, adjusted (for clinical covariates as mentioned above), and adjusted model that excluded BSA.

Statistical significance was accepted for a 2-sided *p* < 0.05. Results of the Cox regression analyses are reported as unadjusted or adjusted hazard ratio (HR) and 95% confidence interval (CI). The statistical analyses were performed with IBM SPSS version 25.0 (Chicago, IL, USA) and RStudio 4.0 (R Foundation for Statistical Computing, Vienna, Austria). 

## 3. Results

The total cohort population comprised 6843 patients (3783 male), the mean age was 76.25 ± 12.6 years, the mean LVEDD was 5.05 (±0.84) (ranging from 4.04 to 9.1 cm), and the mean LVEF was 46.9 ± 11.9% (ranging from 8% to 70%). 

A total of 2413 patients (35.2%) presented with ≥moderate MR. Compared with 4430 patients (64.8%) admitted with ≤mild MR, patients with ≥moderate MR were older, more of the female gender, had lower BMI and BSA, with larger LV end-diastolic dimensions (LVEDD) and lower mean LVEF (Table 1). In addition, this group had a lower rate of comorbidities such as diabetes mellitus, hypertension, COPD, and ischemic heart disease with a lesser frequency of previous myocardial infarction (Table 1). 

**Figure 1:**
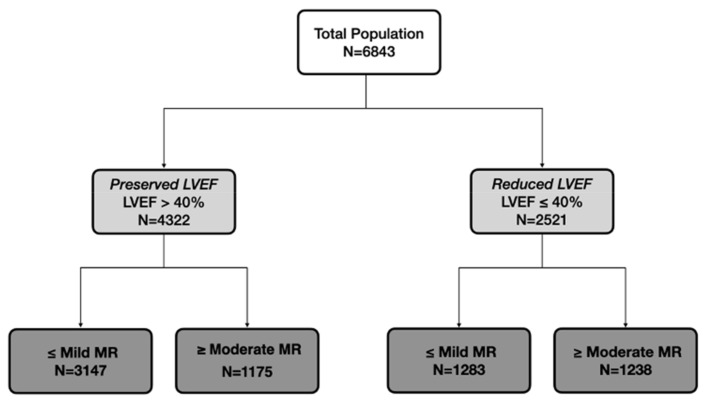
Patient population. LVEF = Left ventricle Ejection Fraction; MR = Mitral Regurgitation.

As patients with LVEF ≥ 50% and patients with LVEF 41–49% behaved similarly (Supplementary Table S1), patients were further divided into two groups according to the LVEF at admission: The preserved LVEF group comprised 4322 patients with an LVEF of >40%, while the reduced-LVEF group comprised 2521 patients with an LVEF of ≤40% (Figure 1). 

Subsequently, each LVEF group was further divided according to MR severity at admission (mild MR or ≥moderate MR), resulting in four subgroups. 

The subgroup analysis is presented in Table 2.

### 3.1. Preserved LVEF (LVEF > 40%) 

The preserved LVEF group comprised 4322 patients; among these patients, 3147 (73%) had ≤mild MR, and 1175 (27%) had ≥moderate MR. 

Preserved LVEF patients with ≥moderate MR were older and more frequently female. Lower rates of diabetes mellitus, hypertension, COPD, and previous MI were noted in these patients.

Preserved LVEF patients with ≥moderate MR were less frequently treated with beta-blockers and ACE/ARB. As the rate of atrial fibrillation/flutter was higher in this group, they were more frequently prescribed oral anticoagulants. 

Preserved LVEF patients with ≥moderate MR had significantly larger left atrial areas (27.5 cm^2^ vs. 23.6 cm^2^; *p* < 0.001) and larger LVEDD (4.89 cm vs. 4.68 cm; *p* < 0.001). More than 80% of patients with preserved LVEF and ≥moderate MR had SPAP above 40 mmHg compared with 55% of the patients with preserved LVEF and ≤mild MR (*p* < 0.001). 

### 3.2. Reduced LVEF (LVEF ≤ 40%)

The reduced-LVEF group comprised 2521 patients. A total of 1238 of these patients (49%) had ≥moderate MR, compared with 1283 (51%) who had ≤-mild MR. 

The reduced-LVEF patients with ≥moderate MR were older, female, with lower BMI, and had a lower LVEF, compared with the reduced-LVEF patients with ≤mild MR. The reduced-LVEF patients with ≥moderate MR had lower rates of diabetes, hyperlipidemia, and IHD with prior MI. A significantly higher prevalence of hypertension, chronic renal failure, and atrial flutter/fibrillation was noted in these patients (Table 2). 

The reduced-LVEF patients with ≥moderate MR had significantly larger left atrial areas (28 cm^2^ vs. 25 cm^2^, *p* < 0.001) and larger left ventricular end-diastolic dimensions (LVEDD, 5.85 cm vs. 5.56 cm) (*p* < 0.001). More than 80% of patients with reduced LVEFs and ≥moderate MR had an SPAP above 40 mmHg, compared with 56% in the reduced-LVEF patients with ≤mild MR group (*p* < 0.001). 

### 3.3. All-Cause Mortality

**Figure 2:**
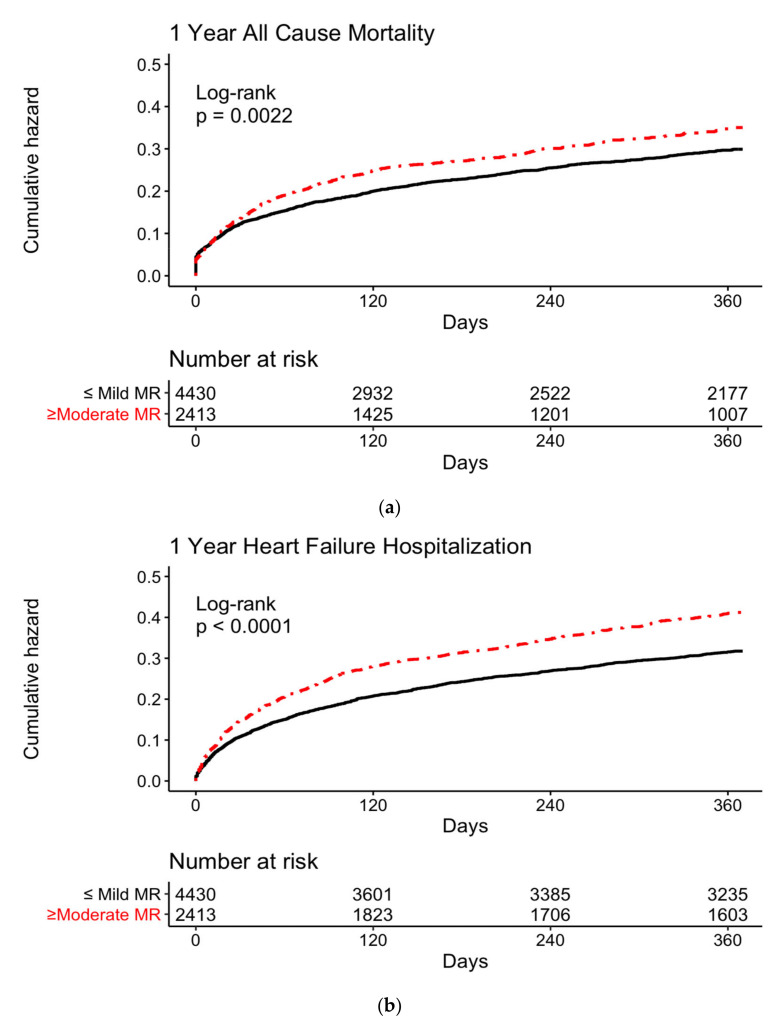
Kaplan–Meier curves for (**a**) 1-year all-cause mortality according to MR severity and (**b**) 1-year recurrent hospitalization according to MR severity.

The one-year post-AHF hospitalization mortality rate in the whole cohort was 23.8%, and patients with ≥moderate MR had a higher mortality rate than patients with ≤mild MR, (25.5% vs. 23.2%, *p* = 0.028), unrelated to the baseline LV function (Figure 2a). 

**Figure 3:**
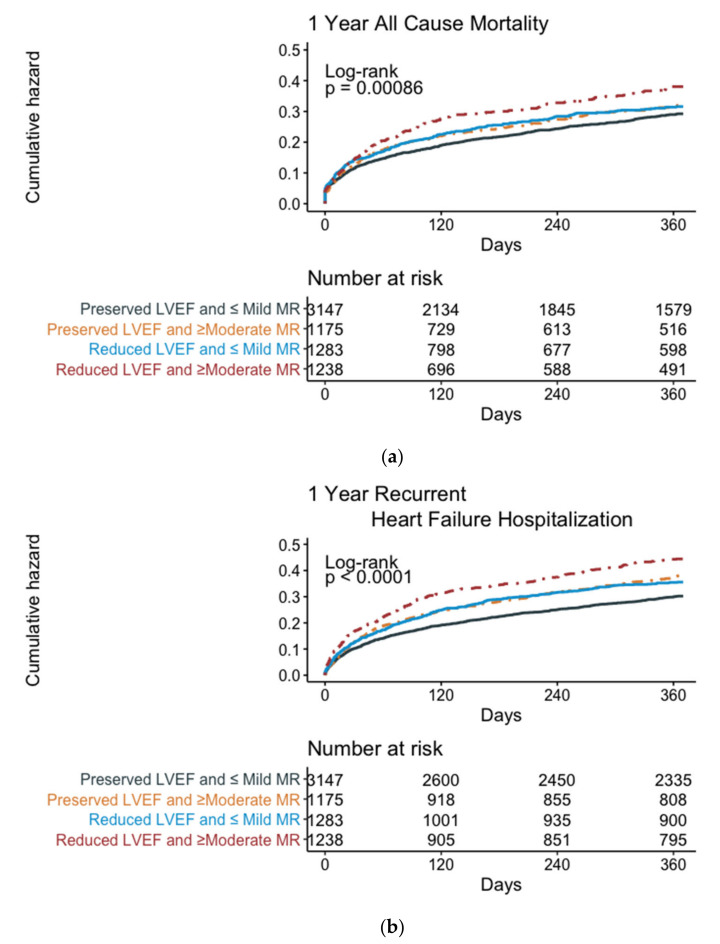
Kaplan–Meier curves for (**a**) 1-year all-cause mortality and (**b**) 1-year recurrent hospitalization according to LVEF and MR severity.

In the group of patients with preserved LVEFs, one-year mortality rates were 22.8% for patients with ≤ mild MR, compared with 24.2% in patients with ≥moderate MR. In the group of patients with a reduced LV function, one-year mortality rates were 23.6% for patients with ≤mild MR, compared with 27.3% in patients with ≥moderate MR (overall log-rank *p* < 0.001, Figure 3a). 

Based on the results of the multivariable Cox regression analysis (preserved LVEF and ≤mild MR as a reference; Table 3, Left) adjusted to age, gender, BSA, atrial fibrillation, IHD, renal failure, anemia, DM, and COPD, the risk of death was increased only in patients with reduced LVEF, irrespective of MR severity (HR: 1.31 [95% CI: 1.12–1.53] for patients with a reduced LV function and ≤mild MR and HR:1.44 [95% CI: 1.25–1.67] for patients with a reduced LV function and ≥moderate MR; *p* < 0.001 for both). After further subgroup analysis, this finding remained true regardless of LVEDD (Supplementary Table S2A).

### 3.4. Rehospitalization Due to Heart Failure

A high rehospitalization rate was noted in the overall cohort, with 29.5% of the patients requiring rehospitalization due to heart failure during the 1-year follow-up. 

In the overall cohort, patients with ≥moderate MR had a higher rehospitalization rate than patients with ≤mild MR (33.8% vs. 27.2%, *p* < 0.001), unrelated to the baseline LV function (Figure 2b).

In the group of patients with preserved LVEFs, 26% of the patients with ≤mild MR required rehospitalization, compared with 31.6% of the patients with ≥moderate MR. 

In the group of patients with reduced LVEFs, 29.9% of the patients with ≤mild MR required rehospitalization, compared with 35.9% of the patients with ≥moderate MR (overall log-rank *p* < 0.001, Figure 3b).

The multivariable Cox regression analysis (preserved LVEF and ≤mild MR as references; Table 3, Right) adjusted to age, gender, BSA, atrial fibrillation, IHD, renal failure, anemia, DM, and COPD showed an increased risk for recurrent hospitalization in each group (Figure 3, Right) (HR: 1.35 [95% CI: 1.17–1.52] for patients with preserved LV function and ≥moderate MR; HR: 1.31 [95% CI: 1.15–1.51] for patients with reduced LV function and mild MR; and HR:1.65 [95% CI: 1.45–1.88] for patients with reduced LV function and ≥moderate MR); *p* < 0.001 for all. After further subgroup analysis, we found an increased risk of rehospitalization in patients with an LVEDD above the median and a reduced LV function, irrespective of MR severity, while in patients with smaller ventricles, the risk of rehospitalization was higher in patients with moderate MR, irrespective of LV function (Supplementary Table S2B).

A total of 64 patients (0.9% of the total cohort) underwent mitral valve replacement during the first year of follow-up comprising 49 patients in the preserved LVEF group, of which 42 (85%) had ≥moderate MR at the index admission. Twenty-two patients in the reduced-LVEF group underwent MVR during the follow-up period, and 17 (77%) of them had ≥moderate MR at the index admission.

## 4. Discussion

The main findings of our observational study are: (1) mortality and morbidity were still very high in this large contemporary cohort of patients admitted with AHF, despite advances in medical management; (2) when comparing patients according to their degree of MR severity during admission, patients with significant (≥moderate) MR had higher mortality rates regardless of LV size; (3) the major independent predictor of mortality was the LVEF; the lower the LVEF, the higher the mortality rate; and (4) significant MR was an independent predictor of rehospitalization.

The interaction between MR and LVEF is complicated since they are intrinsically inter-related. The LVEF is a marker of ventricular remodeling, which is the cause of functional MR owing to LV enlargement and papillary muscle displacement, but functional MR can artificially increase the LVEF through its volume overload [17,18,19,20]. Consequently, extrapolating the independent effect of each variable on the outcome is possible only when large populations, such as those in the current study, are evaluated.

Our study, which evaluated almost 7000 contemporary patients admitted with AHF, reinforces the notion that MR severity is associated with all-cause mortality and extends the current understanding by demonstrating in a large group of patients that mortality is mainly mediated by the baseline LV function [10,21,22,23,24,25]. 

In a similar but much smaller study, Kajimoto et al. [10] evaluated a group of 3557 patients admitted with AHF in 53 hospitals in Japan. In that study, 23.1% of the patients presented with ≥moderate MR, compared with 37% of the patients in the current study, while 43% of the patients had a reduced LV function, compared with 37% in the current study. Similar to our findings, after 530 days of follow-up, MR was associated with a higher risk of rehospitalization but not mortality.

Pecini et al. [26] retrospectively evaluated 3078 patients hospitalized for decompensated heart failure in 43 hospitals in Denmark between 2001 and 2002. Overall, the presence of MR was associated with a worse prognosis. After 4.5 years of follow-up, in patients with an LVEF of <25%, the presence of ≥moderate MR predicted mortality. In that study, it is not clear whether an echocardiographic evaluation was performed during the index admission. 

Mowakeaa et al. [11] previously evaluated the impact of MR severity on the risk of death or heart failure hospitalization in a group of 615 patients with an LVEF of ≤35%. Similar to our findings, they found that MR severity was associated with rehospitalizations but not with mortality. In addition, Kubo et al. [12] showed that even patients who experienced improved MR severity during hospitalization due to AHF had a similar outcome when compared with patients in whom the MR severity did not change during admission and constantly persisted with severe MR. 

The recently published ARIC HF [27] community surveillance study evaluated the prevalence and prognostic significance of MR in acute decompensated heart failure in 3878 patients admitted to four different US community hospitals. In that retrospective surveillance analysis of hospital discharges for heart failure, which included only patients ≥ 55 years old, the presence of ≥moderate MR was associated with an increased risk of mortality in patients with an LVEF of <50%. Overall mortality at 1-year was 30.4%, much higher than that found in the current study. This might be explained by a slightly higher age at admission, as well as a higher prevalence of comorbidities such as hypertension, diabetes, and previous myocardial infarction in that study. Even though the LVEF stratification was different from that in the current study, there were significant differences in the prevalence of significant MR between both studies. While in that study, 44/% of the patients with an LVEF of ≤50% had ≥moderate MR, only 18% of the patients in the current study had reduced LVEFs and ≥moderate MR. This may be at least partially explained by the fact that in ARIC, MR severity was determined by many different readers in different community hospitals, while in the current study, all the echocardiographic studies were evaluated by a team of specialized echocardiographists in a large tertiary center [28]. 

Pagnesi et al. [29] recently retrospectively analyzed the impact of MR in patients with worsening heart failure from both the index and validation BIOSTAT-CHF cohorts [30]. Similar to our findings, patients with ≥moderate MR had larger LV and left atrial dimensions, as well as a lower LV function. Additionally, similar to our findings, after adjustment for clinical and laboratorial variables, ≥moderate MR was not related to total mortality, and the prognostic impact of ≥moderate MR was higher in patients with a reduced LV function. While in the current study, total mortality was related to the LV function regardless of MR or LV dimensions, in the study by Pagnesi, the presence of ≥moderate MR was associated with an increase in the combined end-point of total mortality and hospitalization after 2 years. These differences may be explained by the differences in patient population, follow-up time, and different end-points. The higher mortality rate found in our study (23.8% after 1 year vs. 26.3% after 2 years) may be related to the higher age of our patient population (76 vs. 70 years old) as well as to the fact that 38% of the data evaluated in Pagnesi’s study were acquired from patients evaluated on an outpatient basis, while all our data were derived from patients admitted with acute heart failure. In addition, the echocardiographic assessment in the current study was mostly obtained during the first 2 days of admission, while in Pagnesi’s study, echocardiographic evaluation at recruitment was not mandatory and was performed only when not available during admission [29]. In addition, the current study evaluated a large cohort of consecutive patients admitted to a single tertiary center (44.7% women), while the BIOSTAT-CHF patient population (30% women) was recruited from 69 centers that collaborated in that study. 

Finally, in the current study, patients with enlarged left ventricles and reduced LV functions had a higher risk of rehospitalization irrespective of MR severity, which probably indicates a more long-standing disease with left ventricular remodeling, while the increased risk of rehospitalization found in patients with smaller left ventricles and ≥moderate MR irrespective of their LV function may be explained by a disproportionate MR severity in relation to the LV size, as proposed by Grayburn [31].

## 5. Limitations

The main limitation of this study is its retrospective and observational nature using a single tertiary medical center. Second, the quantitative analysis of MR severity was available in only 1105 patients, but it correlated well with the multiparametric analysis (*p* < 0.001, Supplementary Table S3). These findings are not surprising and are supported by the recent echocardiographic analysis of the COAPT trial, according to which the quantitative analysis of MR severity is not always feasible, and a multiparametric approach, such as the one utilized in this study, is indicated for the proper estimation of MR severity in severe heart failure patients [32]. 

Third, this is a real-world study that evaluated data extending over a 9-year period, and the LVEF was visually estimated as in common clinical practice. As the LV function analysis was dichotomized in two groups (LVEF > 40% or LVEF ≤ 40%), the impact of minor inter-individual variation in the assessment of LV function should not affect the results in a large patient population. Fourth, MR is dynamic in that its severity varies based on loading conditions; however, the main idea behind this study is to assess if MR severity at admission, regardless of the clinical conditions under which it was evaluated, can predict mortality in the long-term follow-up. Fifth, only 0.9% of the patient population underwent mitral valve replacement during the follow-up period, a number that should not affect our findings. We cannot exclude that some patients underwent surgery in a different hospital during this period, but this is very unlikely considering that our medical center is the major surgical referral center in the area. Finally, the reported diagnosis of heart failure was based on the clinical judgment of a treating physician in a real-world tertiary care center. 

## 6. Conclusions

In patients admitted with AHF and undergoing echocardiographic evaluation during hospitalization, the LVEF is the main prognostic determinant for mortality after 1 year. Significant (≥moderate) MR is associated with an increased risk of recurrent hospitalization. 

## Figures and Tables

**Table 1:** Baseline clinical characteristics according to MR severity.

	≤Mild MR (n = 4430)	≥Moderate MR (n = 2413)	*p* Value
Age (Years)	76.1 ± 12.5	76.5 ± 12.7	0.18
Gender (Female)	44.0%	46.0%	0.10
BMI (kg/m^2^)	28.6 ± 5.8	27.2 ± 5.1	<0.001
BSA (m^2^)	1.86 ± 0.47	1.81 ± 0.23	<0.001
LVEF (mean% ± SD)	49.74 ± 14.70	41.70 ± 16.63	<0.001
LVEF > 40% (% of total)	71.0%	48.7%	<0.001
LVEDD (cm ± SD)	4.93 ±1.19	5.39 ± 1.98	<0.001
**Comorbidities**			
Diabetes mellitus	40.2%	33.8%	<0.001
Hypertension	66.9%	58.5%	<0.001
Hyperlipidemia	42.5%	40.1%	0.03
COPD	16.7%	10.6%	<0.001
Anemia	28.5%	27.5%	0.37
Chronic renal failure	34.2%	35.2%	0.4
CVA/TIA	17.1%	14.3%	0.01
Atrial fib/flutter	35.4%	43.1%	<0.001
Current smoker	18.3%	14.6%	0.001
PVD	8.0%	8.0%	0.98
Prior IHD	52.7%	54.5%	0.147
Prior MI	26.1%	24.9%	0.52
Prior cardiomyopathy	6.9%	10.4%	<0.001

Atrial fib = atrial fibrillation; BMI = body mass index; COPD = chronic obstructive pulmonary disease; CRT-D = cardiac resynchronization therapy device with defibrillator; CVA = cerebrovascular accident; ICD = implanted cardioversion device; IHD = heart ischemic disease; LEDD = left ventricular end-diastolic dimension; LVEF = left ventricular ejection fraction; MI = myocardial infarction; PVD = peripheral vascular disease; TIA = transient ischemic accident.

**Table 2:** Baseline clinical characteristics according to LV function and MR severity.

	Reduced LVEF (n = 2521)			Preserved LVEF (n = 4322)		
	≤Mild MR (n= 1283)	≥Moderate MR (n = 1238)	*p*-Value	≤Mild MR (n = 3147)	≥Moderate MR (n = 1175)	*p*-Value
Age (Years)	72 ± 13	73 ± 13	0.05	78 ± 12	80 ± 11	<0.001
Gender (Female)	23.5%	29.9%	<0.001	52.4%	63.1%	<0.001
BMI (kg/m^2^)	27.3 ± 5.1	26.6 ± 4.8	0.01	29.1 ± 6.1	27.9 ± 5.5	<0.001
BSA (m^2^)	1.89 ± 0.63	1.84 ± 0.22	<0.001	1.85 ± 0.39	1.78 ± 0.23	<0.001
LVEF (%)	29 ± 8	27 ± 8	<0.001	58 ± 6	57 ± 7	<0.001
LVEDD (cm)	5.50 ± 0.81	5.81 ± 0.88	<0.001	4.66 ±0.58	4.79 ± 0.64	<0.001
** Comorbidities**						
Diabetes mellitus	42.2%	37.5%	0.05	39.4%	30.0%	<0.001
Hypertension	58.8%	51.4%	<0.001	70.2%	66.0%	0.006
Hyperlipidemia	43.9%	39.7%	0.095	41.9%	40.5%	0.182
COPD	12.9%	9.2%	<0.001	18.3%	12.1%	<0.001
Anemia	22.2%	22.6%	0.89	31.3%	32.7%	0.45
Chronic renal failure	34.8%	37.8%	0.004	49.7%	50.8%	0.66
CVA/TIA	15.0%	12.2%	0.08	18.0%	16.6%	0.50
Atrial fib/flutter	31.1%	34.1%	0.015	37.1%	52.5%	<0.001
Current smoker	24.9%	18.6%	<0.001	15.6%	10.4%	<0.001
PVD	9.7%	10.7%	0.645	7.3%	5.1%	0.013
Prior IHD	69.4%	64.9%	0.017	45.9%	43.6%	0.207
Prior MI	40.9%	33.2%	*p* < 0.001	20.1%	16.3%	0.008
Prior cardiomyopathy	16.9%	16.5%	0.115	2.9%	4.0%	0.202
** Laboratory at Presentation**						
Creatinine	1.56 ± 1.1	1.64 ± 1.0	0.36	1.51 ± 1.2	1.49 ± 1.1	0.96
eGFR (MDRD)	54.7 ± 46.6	51.7 ± 42.6	0.32	53.2 ± 36.6	53.0 ± 39.7	0.8
Hemoglobin	12.1 ± 2.2	11.8 ± 2.1	0.02	11.4 ± 2.1	11.2 ± 1.9	0.013
** Drugs at Presentation**						
Aldosterone blockers	18.9%	19.5%	0.74	12.1%	12.2%	0.93
ACE/ARB	47.1%	42.3%	0.016	47.6%	42.8%	0.005
Beta-blockers	25.7%	21.5%	0.012	39.5%	34.1%	0.001
Loop diuretics	58.4%	57.8%	0.75	40.1%	40.2%	0.97
Oral anticoagulants	18.7%	18.6%	0.93	21.6%	28.3%	<0.001

Values are mean ± SD, n (%). ACE = angiotensin convertase inhibitor; Atrial fib = atrial fibrillation; ARB = angiotensin receptor block; BMI = body mass index; COPD = chronic obstructive pulmonary disease; CRT-D = cardiac resynchronization therapy device with defibrillator; CVA = cerebrovascular accident; ICD = implanted cardioversion device; IHD = heart ischemic disease; LEDD = left ventricular end-diastolic dimension; LVEF = left ventricular ejection fraction; MI = myocardial infarction; PVD = peripheral vascular disease; TIA = transient ischemic accident.

**Table 3:** Adjusted Cox regression analysis for 1-year all-cause mortality (Left) and 1-year year recurrent hospitalization (Right) according to LVEF and MR severity.

	1-Year Mortality			HF Rehospitalization		
Characteristic	HR ^1^	95% CI ^1^	*p*-Value	HR ^1^	95% CI ^1^	*p*-Value
Preserved LVEF and ≤Mild MR	Reference			Reference		
Preserved LVEF and ≥moderate MR	1.01	0.86, 1.17	>0.9	1.34	1.17, 1.52	<0.001
Reduced LVEF and ≤mild MR	1.31	1.12, 1.53	<0.001	1.31	1.15, 1.51	<0.001
Reduced LVEF and ≥moderate MR	1.44	1.25, 1.67	<0.001	1.65	1.45, 1.88	<0.001

Adjusted for age, gender, BSA, IHD, AF, renal failure, anemia, DM, and COPD. ^1^ HR = hazard ratio; CI = confidence interval.

## Supplementary Materials

The following supporting information can be downloaded at: https://www.mdpi.com/article/10.3390/jcm11154267/s1, Table S1: Mortality and rehospitalization rate according to Baseline LVEF and MR Severity; Table S2: Forest Plot–Cox regression analysis for (A) 1-year all-cause mortality and (B) heart failure rehospitalization unadjusted and adjusted according to above or below median LVEDD; Table S3: Correlation Between multiparametrically determined MR severity and effective regurgitant orifice area.

## Abbreviations

MR	Mitral regurgitation
LVEF	Left ventricular ejection fraction
AHF	Acute heart failure
LV	Left ventricle
SPAP	Systemic pulmonary artery pressure
MVR	Mitral valve replacement
LVEDD	LV end-diastolic dimension
LVEDV	LV end-diastolic volume
re-HFH	Recurrent heart failure hospitalizations
