# Supplementary material for: The Prognostic Role of Mitral Valve Regurgitation Severity and Left Ventricle Function in Acute Heart Failure
Source: J Clin Med. 2022 Jul 22;11(15):4267. doi: 10.3390/jcm11154267 (PMC9331219; doi:10.3390/jcm11154267)
Supplement: Supplementary file 1 [file jcm-11-04267-s001.zip › jcm-1791366-supplementary.pdf]

Supplementary Materials_

Supplementary Table S1. Mortality and rehospitalization rate according to Baseline LVEF and MR Severity.

A. LVEF 41-49% vs LVEF ≥50%

1-year mortality *	HR
≤ Mild MR	0.975 CI 95% (0.786-1.209)
≥ Moderate MR	1.085 CI 95% (0.824-1.44)
1-year recurrent hospitalization*	HR
≤ Mild MR	0.939 CI 95% (0.768-1.126)
≥ Moderate MR	1.072 CI 95% (0.846-1.356)

B. LVEF 41-49% vs LVEF ≤40%

1-year mortality *	HR
≤ Mild MR	0.79 CI 95% (0.627-0.9895)
≥ Moderate MR	0.785 CI 95% (0.601-0.98)
1-year recurrent hospitalization*	HR
≤ Mild MR	0.806 CI 95% (0.658-0.988)
≥ Moderate MR	0.881 CI 95% (0.70-1.002)

*Cox multivariable analysis adjusted for age, gender, BMI, renal failure, anemia, diabetes mellitus, and COPD.

Supplementary Table S2. Forest Plot–Cox regression analysis for (A) 1-year all-cause mortality and (B) heart failure rehospitalization unadjusted and adjusted according to above or below median LVEDD.

A. 1-Year All-Cause Mortality

Above Median LVEDD - 4.935cm

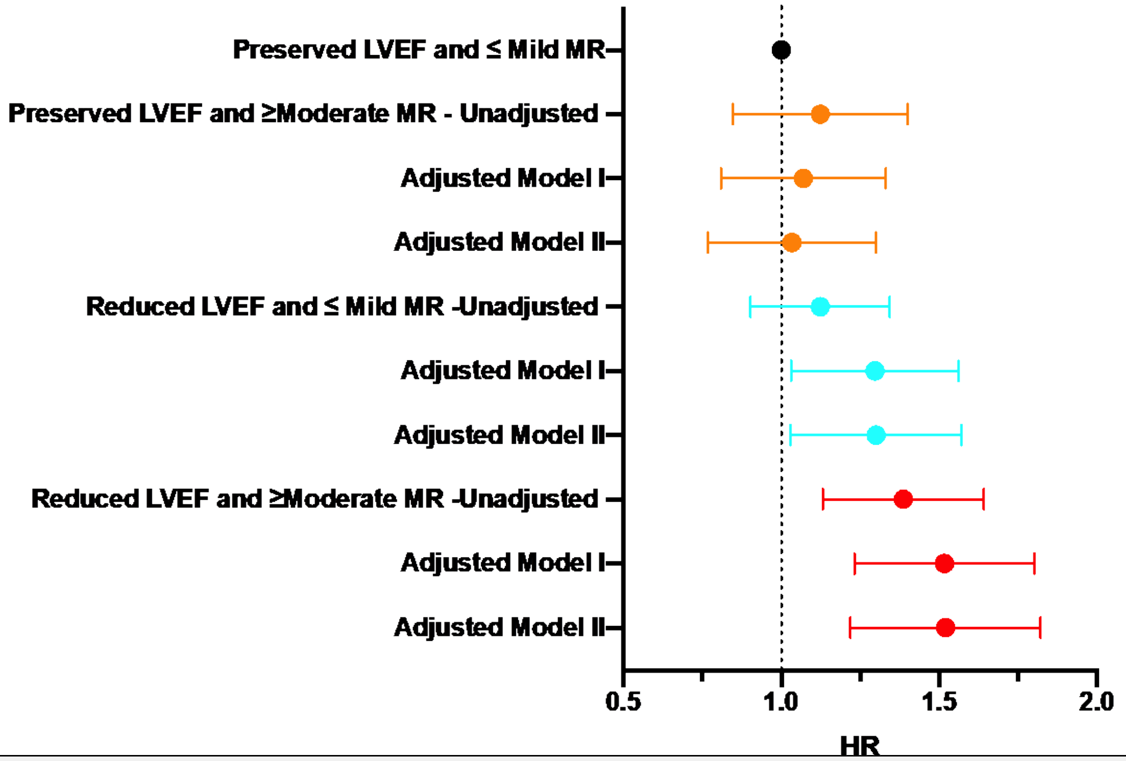

Below Median LV Diastolic

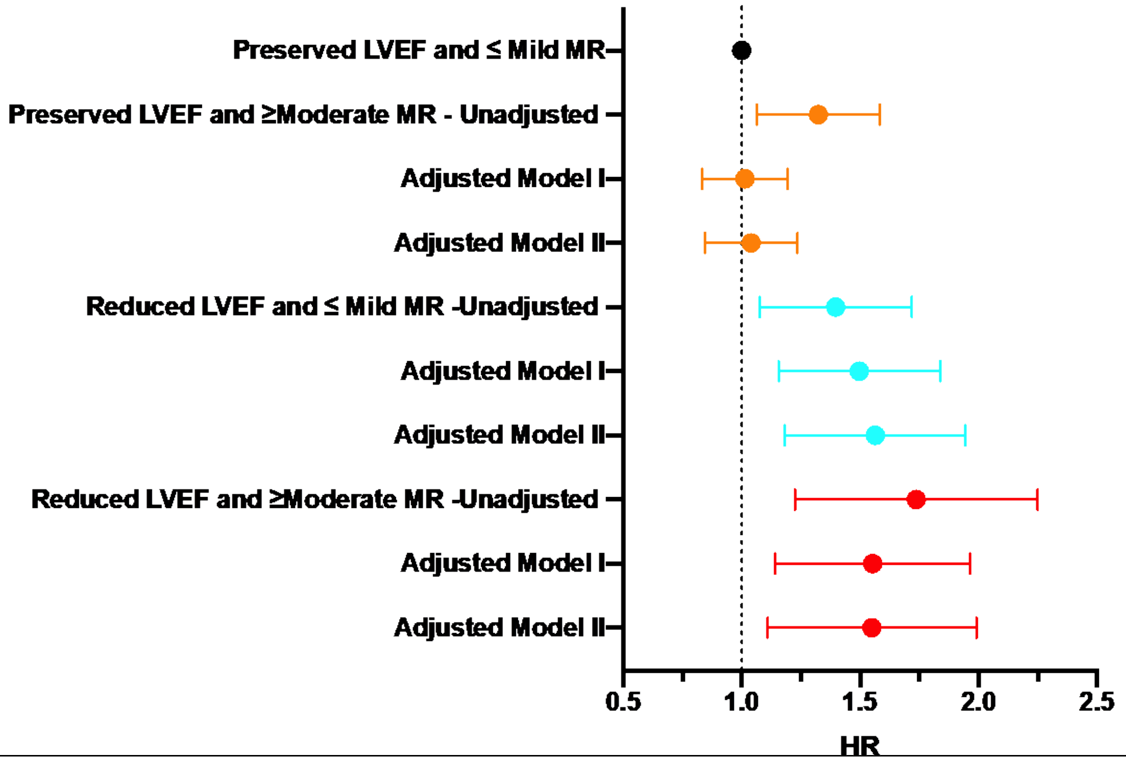

B. Heart Failure Rehospitalization

Above Median LV Diastolic - 4.935cm

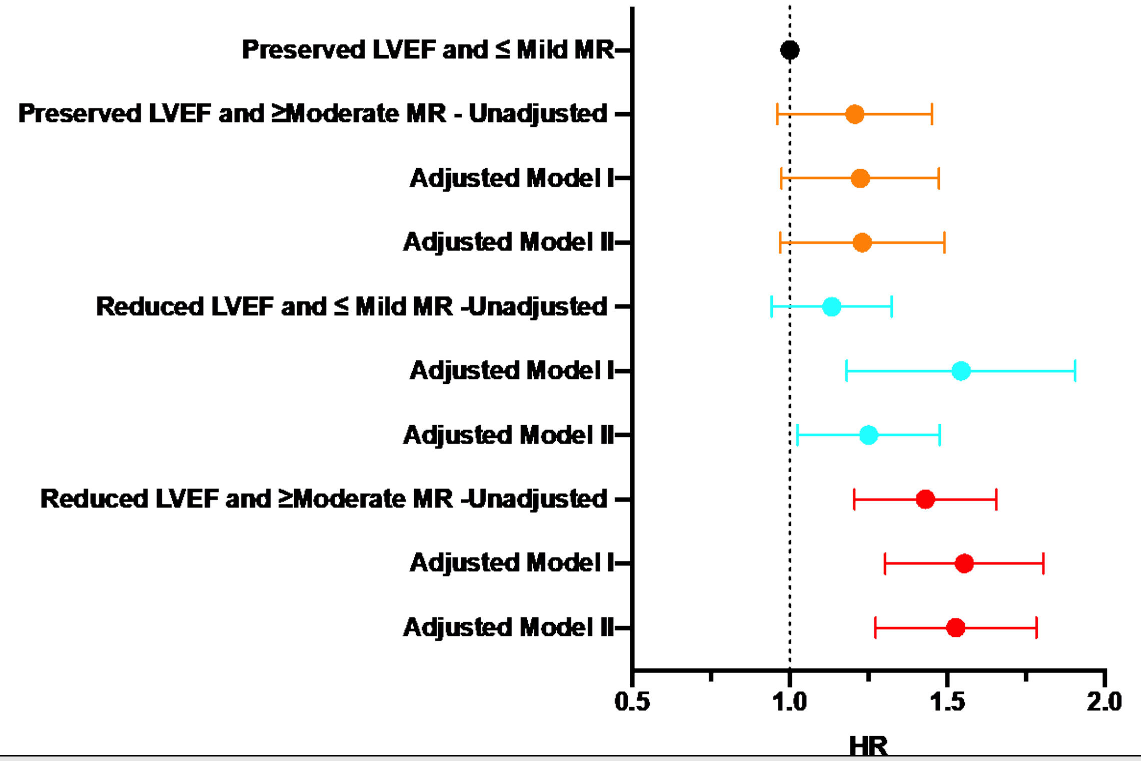

Below Median LV Diastolic - 4.935cm

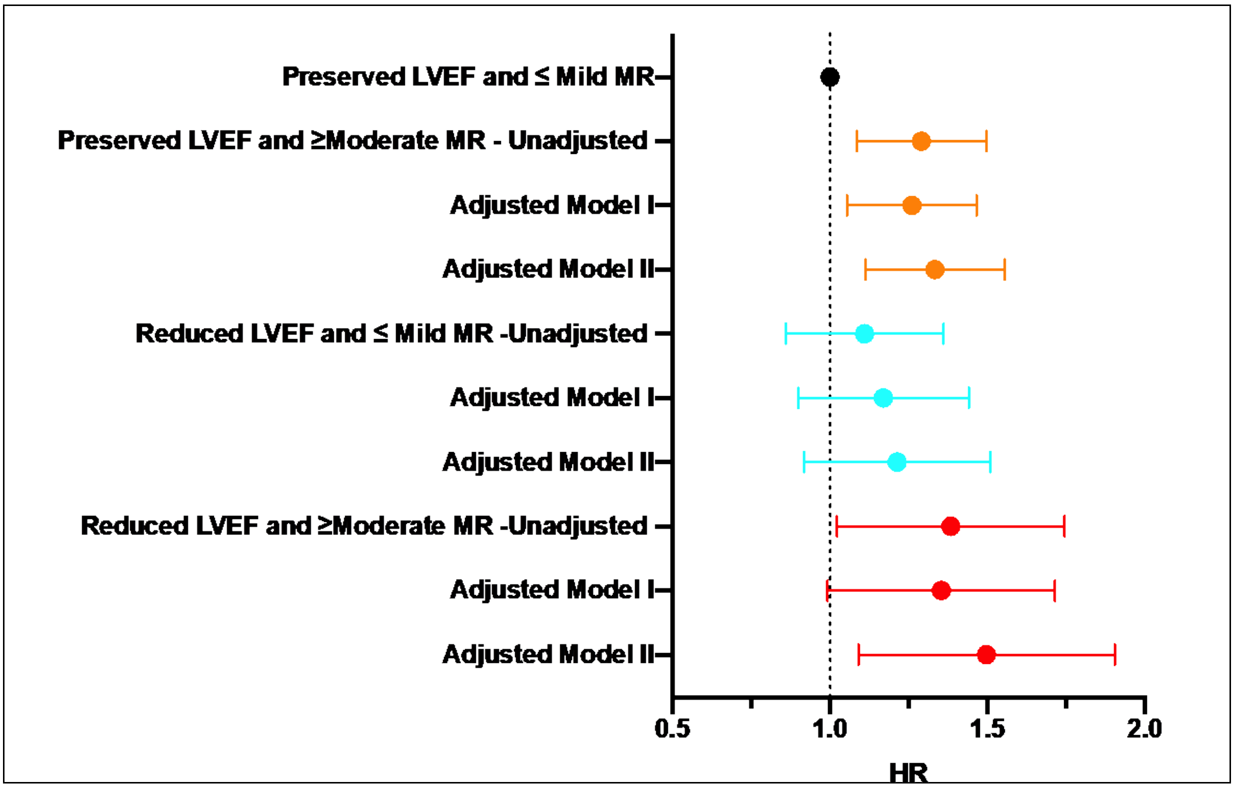

LVEDD = left ventricular end-diastolic dimension.

Adjusted Model 1, age, gender, ischemic heart disease, atrial fibrillation/flutter, renal Failure, anemia, diabetes mellitus,and COPD; Adjusted Model 2, Model 1 + BSA.

Supplementary Table S3. Correlation Between multiparametrically determined MR severity and effective regurgitant orifice area.

	EROA (continuous) n = 1105	P (Person)	EROA (≤ Mild, ≥Moderate) n = 1105	P (Person)
MR Severity – ≤ Mild, ≥Moderate (n = 6843)	0.519	< 0.001	0.296	< 0.001

EROA: effective regurgitant orifice area.
